# The Cancer Aneuploidy Paradox: In the Light of Evolution

**DOI:** 10.3390/genes10020083

**Published:** 2019-01-25

**Authors:** Kristine Salmina, Anda Huna, Martins Kalejs, Dace Pjanova, Harry Scherthan, Mark S. Cragg, Jekaterina Erenpreisa

**Affiliations:** 1 Latvian Biomedical Research and Study Centre, LV1067 Riga, Latvia; salmina.kristine@gmail.com (K.S.); dace@biomed.lu.lv (D.P.); 2 Centre de Recherche en Cancérologie de Lyon, 69008 Lyon, France; anda.huna@gmail.com; 3 Scientific Laboratory of Biomechanics, Riga Stradins University, LV1007 Riga, Latvia; m.kalejs@gmail.com; 4 Bundeswehr Institute of Radiobiology affiliated to the University of Ulm, 80937 Munich, Germany; scherth@web.de; 5 Centre for Cancer Immunology, University of Southampton, Southampton SO16 6YD, UK; m.s.cragg@soton.ac.uk

**Keywords:** cancer, aneuploidy, meio-mitosis, disabled spindle, autokaryogamy, somatic pairing, recombination on kinetochores, reduction, chromothripsis, cleavage embryo

## Abstract

Aneuploidy should compromise cellular proliferation but paradoxically favours tumour progression and poor prognosis. Here, we consider this paradox in terms of our most recent observations of chemo/radio-resistant cells undergoing reversible polyploidy. The latter perform the segregation of two parental groups of end-to-end linked dyads by pseudo-mitosis creating tetraploid cells through a dysfunctional spindle. This is followed by autokaryogamy and a homologous pairing preceding a bi-looped endo-prophase. The associated RAD51 and DMC1/γ-H2AX double-strand break repair foci are tandemly situated on the AURKB/REC8/kinetochore doublets along replicated chromosome loops, indicative of recombination events. MOS-associated REC8-positive peri-nucleolar centromere cluster organises a monopolar spindle. The process is completed by reduction divisions (bi-polar or by radial cytotomy including pedogamic exchanges) and by the release of secondary cells and/or the formation of an embryoid. Together this process preserves genomic integrity and chromosome pairing, while tolerating aneuploidy by by-passing the mitotic spindle checkpoint. Concurrently, it reduces the chromosome number and facilitates recombination that decreases the mutation load of aneuploidy and lethality in the chemo-resistant tumour cells. This cancer life-cycle has parallels both within the cycling polyploidy of the asexual life cycles of ancient unicellular protists and cleavage embryos of early multicellulars, supporting the atavistic theory of cancer.

## 1. Introduction

*‘Nothing in biology makes sense except in the light of evolution.’ (T. Dobzhansky, 1973)*

[1].

Accumulating evidence shows that cancer is associated with genome doubling events that correlate with increasing chromosomal instability and aneuploidy. This process is greatly enhanced by loss of TP53 tumour suppressor function [2] and favours tumour progression, leading to poor prognosis [3,4,5]. However, chromosome instability and associated breakage-fusion-bridge cycles and aberrant mitoses resulting in aneuploidy should ultimately impair tumour proliferation, thus rendering the ‘aneuploidy paradox’. Moreover, chromothripsis, whereby seemingly catastrophic breakage and re-assortment of the genome and its chromosomes occurs, has been observed in tumour genomes [6,7,8]. Chromothripsis with its shattering of partial genomes in one non-clonal event is considered a witness to the genomic chaos present in tumours before repair-mediated rescue [9,10].

Furthermore, the increasing gene mutation load observed in most tumours may also impede tumour development, even if ‘cancer is beyond speciation’ [11]. How then can this paradox be resolved? As often, clues can be found in the distant past. A similar predicament was present long ago during evolution for asexual protists. In the absence of sexual reproduction it is predicted that asexual protists should accumulate harmful mutations through ‘Muller’s ratchet’ [12,13] and become extinct as a species. However, numerous protists have persisted for aeons, indicating the presence of compensatory mechanisms that counteract the accumulation of deleterious gene mutations.

**Figure 1:**
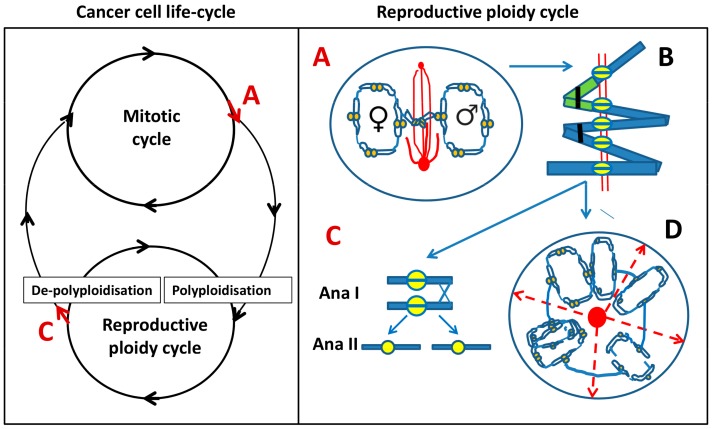
**Left**: Model of cancer cell life cycle that is composed of two reciprocal cycles: mitotic and reproductive ploidy cycle. Red arrows at **A** and **C** denote asymmetric cell fate decisions, indicating potential exit points for each of the two cycles. **Right**: The components of reproductive ploidy cycle: **A**—a cell enters pseudo-mitosis (PM) and segregates replicated parental genomes with end-to-end linked chromosomes and mitosis-uncoupled spindle, lying perpendicular to the axis of the two chromosome groups. **B**—scheme of the end-to-end linked inversely oriented bivalents (depicted as a green–blue-stained pair) and representing a tandem chain of end-to-end linked genome dyads in an endoprophase, a positioning that can allow both zig-zag and co-parallel bi-valent orientation. Homologous bivalents are connected at subtelomeric regions (black clips); recombination is likely occurring between sister centromeres (yellow split rings). AURKB likely provides the microtubule attachment and prevents separation of cohesed centromeres at a monopolar spindle. **C**—linear de-polyploidisation by bipolar meiosis I-like reductional division (Anaphase I), followed by a second Anaphase II division (omitting S-phase), returning the cell into a mitotic cycle. **D**—alternative variant of de-polyploidisation, in which a cell has passed from **A** and **B**, through karyotomies using semi-spindles, and bipolar and multipolar spindles with dicentric bridges, including possible re-assortings by pedogamic fusions of the neighbouring karyotomy products and subsequent release of cellularised sub-nuclei through radial cleavage furrows (dashed red arrows), originated from a fused centrosome-containing mid-body (red circle). The released cell(s) may again enter the reproductive cycle, or even a mitotic cycle, possibly through an intermediate sub-generation.

Early unicellular organisms of this type are an interesting comparator, because tumours also shift their genome expression towards the gene phylostrates of pro- and lower eukaryotes [14,15,16], particularly when it comes to chemoresistance, as seen by single cell transcriptome analysis [17]. Moreover, resistant metastatic human cells acquire phenotypes of single-cell eukaryotes with amoeboid features [18,19]. Our previous studies revealed that the cancer epigenome shifts towards enhanced expression of unicellular genes, which is associated with genome duplications and accompanied reprogramming [20,21,22]. In the absence of ‘Muller’s ratchet’, it is supposed that the polyploidy that lower protists exhibit allows them to utilise gene conversion for mutational correction [23]. In turn, we came to the conclusion that reversible polyploidy with its associated reprogramming reinstates the tumour germ line, operating as a reproductive cycle in a reciprocal relationship with a proliferative mitotic cycle. In the most general form, this idea finds its parallels in the life-cycles of protozoa with cycling polyploidy [24,25] and has been formulated as a ‘cancer life cycle’ (Figure 1, left; modified from [26,27]). Transient, reversible polyploidy was shown to utilise certain meiotic features and related aspects of endomitosis [28] (as defined by Geitler [29]), which may have preceded the evolution of zygotic and gametic meiosis [30].

Transient, reversible polyploidy in cancer cells seems to depend on the expression of the meiotic kinase Mos [27,28,31,32,33]. Accordingly, the expression of meiotic genes has been noted in many tumour cells, both primary clinical material and cell lines [32,33,34,35,36,37,38,39,40] and found to be predictive of poor prognosis. In particular, the expression of Mos, REC8, SGO1,2, SPO11, DMC1, RAD51, SCP1, SCP3, STAG3, HORMA, and many other, so-called cancer testes-associated antigens has been observed. However, the relationship of canonical meiosis to a currently designated ‘meio-mitosis’ in somatic polyploid cancer cells remains unresolved [41]. Moreover, the evolution of meiosis and sexual reproduction are still the subject of ongoing discussions [42,43,44,45,46,47,48,49]. Recently, protistologists have paid attention to the similarity of amoebal life-cycles, including the polyploidy phase and the return to generative diploidy or haploidy, with the ‘life-cycles’ of resistant tumour cells [50,51,52]. However, the significance of meiotic gene expression in asexual amoebas themselves is currently not understood [52,53] and may need re-evaluation [54].

With respect to potential cellular mechanisms, in our previous studies, we used a p53-mutant cancer model, in which lymphoma cells were exposed to a single 10 Gy dose of ionising irradiation (IR) [55]. Subsequently, cells entered a prolonged G2/M arrest and underwent increasing polyploidy reaching 25–30% of population that was accompanied by massive apoptotic crisis leaving alive 5–6 days post IR <10% of these giant cells. Further, a proportion of the survived endopolyploidy cells underwent sub-nuclear sorting and de-polyploidisation, which led to the recovery of a clonogenic mitotic fraction 2–3 weeks post-insult. FACS analysis and 24 h DNA pre-labelling with 3H-Thymidine showed that repopulation of the culture originated from the polyploid (>4C) fraction. This sequence of events also seen in doxorubicin-treated cells [55] was dependent on dysfunctional TP53. Re-irradiation of the recovered TP53-deficient clones (taken on 40 days post first IR) resulted in similar radioresistance curves and dynamics of polyploidy and apoptosis as the original cells, arguing against the selection of an existing resistant sub-clone. Notably, the recovered fraction in both cases was equally small (<0.01%). These data indicate the leading role of reversible polyploidy after genotoxic DNA damage for survival. In this model, fluorescence in situ hybridisation (FISH) analysis of centromeric labels of four chromosomes (#1, 3, 4, 9) revealed aneusomy or nullisomy in 30% of polyploid giant cell subnuclei, while the recovered fraction was para-diploid by DNA content [24]. Somewhat similar results were obtained by Vitale and colleagues [33] treating p53^-/-^ HCT 116 colon cancer cells with 100 nM nocodazole for 48 h with a two-week follow up. The cells polyploidise; an isolated ~8C fraction was clonogenic in vitro and tumorigenic in vivo and finally produced the ~2C population. However, the intermediate sub-4C population was initially highly aneuploid (tested by cen-FISH of #8, #10, and #18), but aneuploidy decreased from 94 to 19% before final depolyploidisation. Repeated treatment of the tetraploid fraction led to the same result. The process of recovery was Mos-dependent. In contrast, experiments by Niu and colleagues [56] using high dosage paclitaxel on ovarian cancer reached ~90% of polyploidy by Day 14 and, using SKY FISH, found highly karyotypically re-arranged para-diploid daughter cells two weeks later.

In an attempt to reconcile the aneuploidy paradox and assimilate all of the data detailed above, here we report on certain mechanisms of ‘meio-mitosis’ observed in various cancer cell radio/chemotherapy models, searching for similarities associated with the meiotic process (found in rare cells in each model). We aim to present a model incorporating elements of cycling polyploidy and cancer cell life cycles and recognising the established relationship of treatment resistance with reversible genome doubling and reprogramming [33,55,57,58,59,60,61,62,63,64,65,66]. Using this assembled material, we provide new evidence for the presence of a peculiar asexual life-cycle operating through reversible polyploidy in cancer cells whose elements might have appeared during the evolution of eukaryotes, far before canonical gametic meiosis developed.

## 2. Materials and Methods 

### 2.1. Cell Lines

The Burkitt’s lymphoma (Ramos and Namalwa), ovarian teratocarcinoma (PA1), and HeLa cell lines were obtained from the ATCC and breast adenocarcinoma MDA-MB-231 cells from ECACC. The lymphoblastoma WIL2-NS was obtained from Dr P. Olive (Canada). Lymphoma cell lines (Namalwa, Ramos, WIL2-NS) were maintained in RPMI-1640 containing 10% foetal bovine serum (FBS; Sigma-Aldrich, St. Louis, MO, USA) at 37 °C in a 5% CO_2_ humidified incubator without antibiotics. PA-1 and MDA-MB-231 cells were cultured in Dulbecco’s modified Eagle’s media (DMEM) supplemented with 10% FBS, and HeLa S3 cells in HAM-1 (Sigma-Aldrich, St. Louis, MO, USA) medium were supplied with 10% FCS. For some experiments, HeLa and MDA-MB-231 were cultured on cover- or chamber-slides.

### 2.2. Cell Treatments

For experimental studies, cells were maintained in a log phase of growth and treated (lymphomas) with a single acute 10 Gy dose of γirradiation (1–2 Gy/min, Clinac 600 C, Varian Medical Systems or HeLa) using a Gulmay D3 225 X-ray source at a dose rate of 0.77 Gy/min), 8 µM ETO (etoposide; Sigma-Aldrich, St. Louis, MO, USA) for 20 h, 100 nM DOX (doxorubicin) for 24 h, or 30 or 100 nM PXT (paclitaxel, Ebewe Pharma, Unterach am Attersee, Austria) for 20 h. After irradiation or drug removal, cell cultures were maintained by replenishing culture medium every 2–3 days and sampled over a 2–3 week period post-treatment. To determine the capacity of cells to replicate DNA, BrdU was added at 5 µM to the cell culture 20 h prior to sample collection. In the experiments with REC8 detection, 10 μM lactacystin was added to the culture medium 2 h before cell harvest.

### 2.3. Immunofluorescence

Cells were pelleted, suspended in warm FBS and cytospun onto glass slides or were grown on chamber slides. Cytospins were fixed in methanol for 7 min at −20 °C, dipped 10 times in ice cold acetone, and allowed to dry. Slides were then washed thrice in TBS 0.01% Tween 20 (TBST) for 5 min. Slides were subsequently blocked for 15 min in TBS, 0.05% Tween 20%, and 1% BSA at room temperature. Samples were covered with TBS, 0.025% Tween 20%, 1% BSA containing primary antibody, and incubated overnight at 4 °C in a humidified chamber. Samples were then washed thrice in TBST and covered with TBST containing the appropriate secondary antibodies goat anti-mouse IgG Alexa Fluor 488 (A31619, Invitrogen, Carlsbad, CA, USA) or sheep anti-human-IgG-FITC or goat anti-rabbit-IgG Alexa Fluor 594 (A31631, Invitrogen) and incubated for 40 min at room temperature in the dark. Slides were washed thrice for 5 min with TBST and once for 2 min in PBS. Samples were then counterstained with 0.25 μg/mL DAPI for 2 min or with propidium iodide (5 mg/mL), and finally embedded in Prolong Gold (Invitrogen). When staining for tubulin, the drying step was omitted. Fixation in methanol/acetone (1:1) was performed, and detergent was absent from all buffers. PF fixation was applied. For BrdU staining DNA denaturation was performed with 2 N HCl, 37 °C, for 20 min before blocking. Primary antibodies and their source are listed in Table 1. For microscopic observations, a fluorescence light microscope (Leitz Ergolux L03-10, Leica, Wetzlar, Germany) equipped with a colour video camera (Sony DXC 390P, Sony, Tokyo, Japan) was used. To capture fluorescent images, in addition to separate optical filters, a three-band BRG (blue, red, green) optical filter (Leica, Wetzlar, Germany) was used. Confocal microscopy was carried out using a confocal laser microscope (Leica SP8 Confocal, Leica, Wetzlar, Germany).

### 2.4. Toluidine Blue DNA Staining and Image Cytometry

Cytospins were prepared and fixed in ethanol/acetone (1:1) for >30 min at 4 °C and air-dried. Slides were then hydrolysed with 5 N HCl for 20 min at room temperature, washed in distilled water (5 × 1 min), and stained for 10 min with 0.05% toluidine blue in 50% citrate-phosphate McIlvain buffer pH 4. Slides were rinsed with distilled water, blotted dry, and dehydrated by incubating twice in butanol for 3 min each at 37 °C. Samples were then incubated twice in xylene for 3 min each at room temperature before being embedded in DPX. Digital images were collected using a Sony DXC 390P colour video camera calibrated in the green channel. DNA content was measured as the integral optical density (IOD), using Image-Pro Plus 4.1 software (Media Cybernetics, Rockville, MD, USA). The stoichiometry of DNA staining was verified using the values obtained for metaphases compared with anaphases and telophases (ratio 2.0); arbitrary diploid (2C) DNA values were averaged from measuring anaphases in non-treated tumour cells; the sum method error was estimated to be less than 10%. For morphological purposes, we used the same reaction, shortening hydrolysis with 5 N HCl to only 1 min.

### 2.5. Fluorescence In Situ Hybridisation (FISH)

Cells were harvested, washed with warm PBS, treated with 75 mM KCl at room temperature for 10–30 min, and fixed with five changes of fresh methanol/glacial acetic acid (3:1). The suspension was dropped (or in some experiments cytocentrifuged) onto slides and allowed to dry. FISH for X and Y (XCE X/Y, D-0825-050-OG, Meta Systems, Altlussheim, Germany) and chromosome 18 (mFISH paint, Meta Systems, Altlussheim, Germany) was carried out using pepsin pretreatment [67], followed by a denaturation step for 5 min at 75 °C and hybridisation at 37 °C overnight. Denaturation and hybridisation steps were performed on a ThermoBrite programmable temperature controlled slide processing system. Slides were mounted in an antifade solution (Vector Laboratories, Burlingame, CA, USA) or in Prolong Gold with DAPI (Invitrogen).

### 2.6. Electron Microscopy

For electron microscopy (EM), cells were fixed in 3% glutaraldehyde in 0.1 M cacodylate buffer, pH 7.2, containing 1 mM CaCl_2_, washed in this buffer with 0.23 M sucrose, postfixed in 2% osmium tetroxide in cacodylate buffer and 2% uranyl acetate in distilled water, dehydrated, and embedded in Spurr resin. Ultrathin sections were contrasted with lead citrate.

## 3. Results

### 3.1. Paired-Group Chromosome Segregation by Pseudo-Mitosis in Genotoxically Challenged Tumour Cells

**Figure 2:**
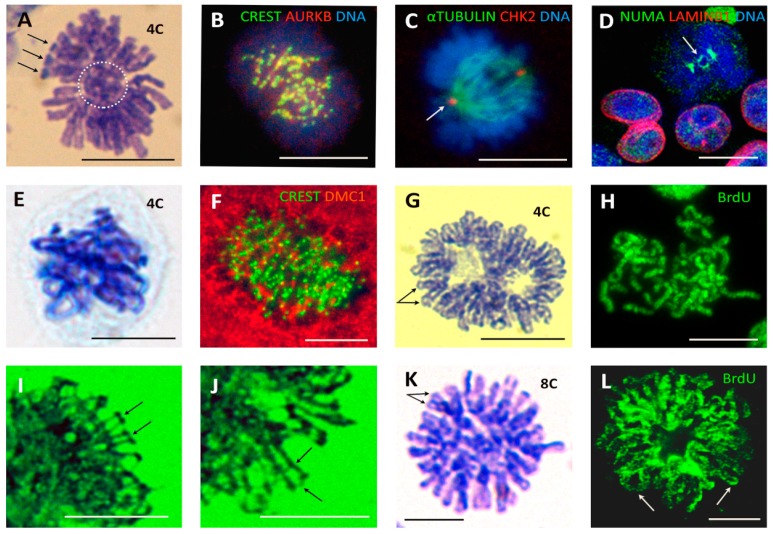
The non-conventional cell division patterns creating polyploidy: 4C PM segregating two ‘buttoned’ groups of dyads and 8C ‘bi-looped metaphases’ (BLM), observed in untreated germ-cell cancers and various somatic tumour cell lines and enhanced after genotoxic or spindle insults. (**A**–**D**) PM in the non-treated ovarian embryonal carcinoma PA1: (**A**) Two separated groups of bi-nemic chromosomes with connected telomere ends (arrowheads) and a central chromosome ‘button’ (dashed circle) in a cell with 4C DNA content (determined cytometrically, for details, see Methods). (**B**) Two groups of dyads in a PM showing centromere doublets (green CREST stain for kinetochores) colocalising with AURBK (yellow). (**C**) A misoriented spindle (with an asymmetrically stronger left pole) aligned perpendicularly to the axis of two closely opposed chromosome groups. (**D**) In PM, NUMA encircles the central DNA ‘button’ (arrow) connecting the two opposed chromosome groups. (**E**) Tetraploid PM (confirmed cytometrically) composed of two laced, not fully separated groups of end-to-end linked chromosomes with closely cohesed chromatids (HeLa cell, 19 h after 10 Gy IR). (**F**) A large metaphase-like breast cancer MDA MB 231 cell on 7 days after doxorubicin (DOX) treatment, with a characteristic composition of tandem sets of centromeres (kinetochores, green), interspersed and partly colocalised with DMC1 (red). (**G**–**J**) Mimic of PM by inactivation of spindle with paclitaxel (PXT) treatment of WIL2-NS lymphoblastoma cells. (**G**) Two binemic chromosome groups with closed telomeres are seen forming two rings in a 4C cell 44 h after PXT (30 nM) treatment. (**H**) BrdU-labelled (20 h) PM cell showing two groups of laced chromosomes with both chromatids labelled, indicating missed mitosis. (**I**,**J**) Two enlarged details of a similar polyploid metaphase-like figure after 72 h of PXT (100 nM) treatment showing fine DNA bridges between sister telomeres and between sister and other arms indicative of recombinogenic DNA repair. (**K**,**L**) Bi-looped metaphase-like figures in WIL2-NS cells 5 days after 10 Gy: (**K**) (DNA measured) and (**L**) incubated with BrdU for 20 h and sampled on Day 8. Bars equal 10 µM.

The wt TP53 ovarian cancer cell line PA1, which possesses a diploid karyotype and the expression profile and phenotype of embryonal carcinoma [20,68], can be considered a model of a cancer stem cell. Therefore, we examined this model in chemoresistance studies. Non-treated PA1 cells perform two types of divisions—conventional mitoses (CM) with a bipolar spindle segregating sister chromatids and, in about 12% of cells, pseudo-mitosis (PM) involving metaphase-like figures separating two groups of bi-nemic chromosomes that are interlaced or ‘buttoned’ together (Figure 2A). Both types of mitoses contain the same amount of DNA (4C as measured by DNA cytometry; *n* = 50).

The centromere doublets of the CM are colocalised with AURKB in a centromeric ring at metaphase and monocentric chromatids segregate during anaphase, while AURKB transits onto the central spindle, enabling karyokinesis (not shown). Contrary to this, the doubled centromeres of PM are both colocalised and tandemly arrayed by AURKB in two, largely discrete, semi-circles (Figure 2B), with AURKB not transiting to the spindle microtubules. In PM, the spindle is oriented perpendicularly to the axis of the pair-grouped chromosome segregation, and is defective; often asymmetrically stronger at one pole (Figure 2C, arrowed).

Next, we stained CM and PM for NUMA expression. NUMA is the nuclear matrix material that first accumulates centrally during CM before its separation to the anaphase poles. In PM, the central DNA ‘button’ is encircled by NUMA, connecting the two chromosome groups (Figure 2D). PMs in PA1 cells consistently displayed connected sister telomere ends (Figure 2A). These PM features—connected sister telomeres, aberrant axis orientation of the mitotically inactive spindle (disabling amphitelic kinetochore attachment and karyokinesis), and NUMA connecting the two chromosome groups—impair both mitotic spindle function and the separation of sister chromatids in PM. Instead, there are faulty bi-kinetochore-labelled centromeres in the two mitotically inactive chromosome groups per tetraploid cell.

While in normal PA1 cells CM segregates the diploid genetic material into two 2C, 2n daughters, PM forms a tetraploid bi-nuclear or bi-lobed 4C, 2n nucleus. We also studied the chemoresistance of PA1 cells in experiments with etoposide (ETO) [68,69]. In general, during recovery of the self-renewing stemline (sampled on Days 7–14), the proportion of CM and PM approximated the control levels but in one experiment during the 3rd week post-IR, we observed highly stringent cell divisions with a threefold increase in A + T/M ratio in CM, where the proportion between CM and PM was shifted in favour of the doubled number of PM (Table 2). These observations suggest that both division types are involved in the recovery of cancer stem cells from genotoxic insults.

Pseudo-mitosis with similar morphologic characteristics were also found in irradiated HeLa cells (Figure 2E), lymphoma cell lines (not shown), and MDA MB 231 breast cancer cells, both in control, untreated populations (1%) and more prevalently in doxorubicin-treated cells (up to 10%) (Figure 2F).

To mimic PM, we undertook a series of experiments with the male para-diploid lymphoblastoma cell line WIL2-NS treated with low doses of paclitaxel (PXT), which inactivates the mitotic spindle and induced typical 4C PM (Figure 2G). Hypotonic spreading revealed chromosomal twin rings with approximately haploid (~23) chromosome numbers in each of the two binemic groups, in contrast to the doubled number of uninemic chromosomes that would be expected in a mitotic division. Testing them for BrdU inclusion, we obtained bi-chromatid labelling (Figure 2H) indicating omission of cell division in tetraploid cells. Furthermore, replicated chromosomes were linked end to end in the two separated groups of tetraploid PMs (Figure 2H), akin to what was seen in the PM of irradiated tumour cells (Figure 2E). Interestingly, the telomere ends were closely associated or fused and remained so even 48 h or longer after PXT treatment. In some metaphase-like figures at 72 h, we detected fine DNA bridges between sister telomeres and between sister arms (Figure 2I,J) indicative of recombinogenic DNA repair. These observations suggest that PM with a mitotically dysfunctional spindle is likely a feature of a slowly cycling subpopulation of cancer stem cells, whose frequency is enhanced by genotoxic treatments and may contribute towards the genotoxic resistance of tumour cells.

### 3.2. Double-Looped Metaphase-Like (DLM) Cells Display Features of the Meiotic Endo-Prophase

**Figure 3:**
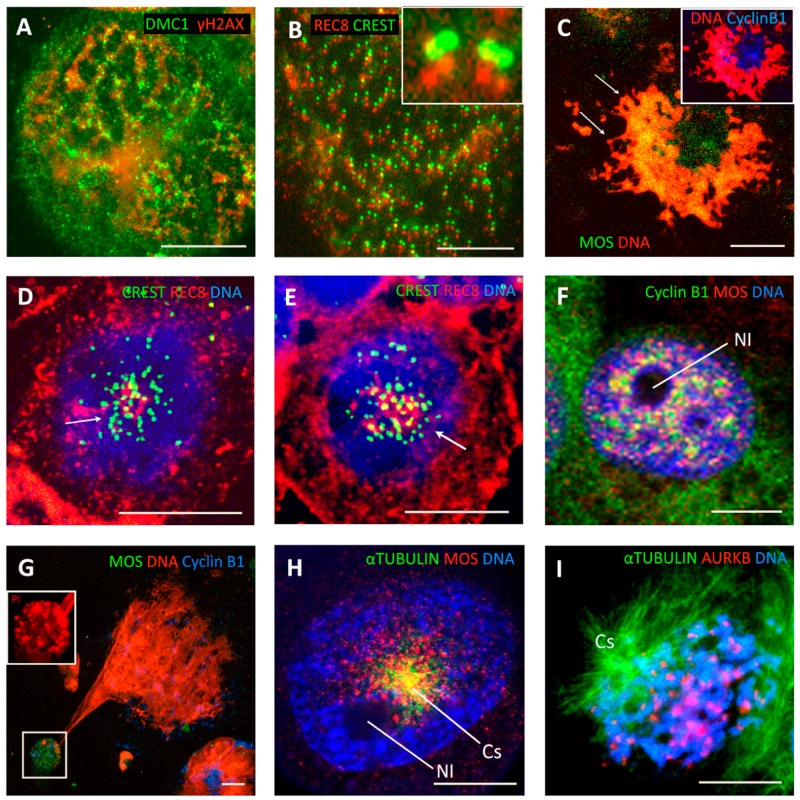
Features of monopolar meiotic endo-prophase in treated tumour cells. (**A**) WIL2-NS cell 6 days post 10 Gy, showing a looped pattern of DNA strands (inferred from chains of tandemly situated foci of DMC1 (red) and γ-H2AX (green) indicating homologous recombination repair of DNA DSB. (**B**) Detail of a WIL2-NS cell nucleus, 5 days post 10 Gy, displaying tandem arrays of kinetochore doublets (green) colocalised with REC8 (red). The boxed and enlarged insert is a typical pattern of kinetochore doublets and REC8 signals. The red and green foci are offset to better demonstrate their colocalisation (reproduced with permission [38]). (**C**) Confocal section of a Namalwa lymphoma cell (6 days post 10 Gy), showing a central location of MOS (green) and CYCLIN B1 (blue). DNA is stained red by propidium iodide. (**D**,**E**) Confocal sections of WIL2-NS cells (5 days after 10 Gy), showing a single cluster of centromeres (arrow) colocalised with REC8. (**F**) The association of MOS (red) and CYCLIN B1 (green) with the perinucleolar rim (Nl: nucleolus) and their radial strands in a MDA MB 231 cell (2 days after DOX treatment). (**G**) The polar cluster of heterochromatin (boxed; DNA (red) stained with 7-AAD) colocalised with MOS (green) CYCLIN B (blue) in a late endo-prophase. (**H**,**I**) Monopolar spindle in a late endo-prophase in MDA MB 231 DOX-treated cells. (**H**) Centrosomal monoastral MTs (green) with granular MOS signals (red) on microtubules radiating from near the nucleolus (Nl), (4 days post DOX-treatment). (**I**) A monopolar (MTs green) diakinetic endo-prophase revealing largely tandem radial arrays of AURKB (red) positive kinetochore doublets on chromosome pairs (DNA blue) in a 3D-preserved sample. Bars equal 10 µM.

Five to eight days post IR in lymphoma WIL2NS and Namalwa cell lines, rare double-looped metaphase-like (DLM) 8C DNA figures appeared among the prevailing polyploid population (Figure 2K). Twenty-hour-long incubation with BrdU disclosed fully labelled radially extending single and loosely interlaced double loops, indicating a second round of DNA endo-replication (Figure 2L) (however, conventional one-chromatid BrdU labelling with open chromatid ends was also encountered in the mitoses of the same samples). By virtue of their closed chromosome ends and their inclusion of BrdU in both chromatids, the 8C DLM cells were similar to 4C PM (Figure 2A,E,G,H) and could have derived from the former. These DLM-like figures appeared mono-polar. The irradiated cells, with the DNA loops emanating from one polar centre, contain tandem DMC1/γH2AX foci indicating that meiotic type homologous recombination is occurring in them (Figure 3A) and could be classified as a stage of meiotic prophase. In turn, the colocalisation of the kinetochore tandem doublets with the doublets of REC8 of the long DNA threads has been described earlier in the polyploid ‘interphase’ cells, indicative of meiotic type recombination in these foci (Figure 3B). To this end, the dense centromere/kinetochore tandem arrays partly colocalised with the meiotic recombinase DMC1 were also found in the polyploid PMs of DOX-treated MDA MB 231 cells (Figure 2F). Collectively, these findings suggest that the centromere/kinetochore doublets persisting from 4C PM to 8C DLM figures are directly engaged through REC8/DMC1/DNA double-strand breaks (DSB) in meiotic-type recombination DNA repair (tandem arrays of RAD51/γH2AX in these cells have also been found [38]. It follows that both unusual figures, PM and DLM, are components of this modified meiotic process. Further remodelling of DLM figures was found associated with the meiotic kinase, MOS. Together with CYCLIN B1, MOS appeared in the centre of the DLM-like figures and less abundantly around the DNA loops (Figure 3C). In the nuclei of these IR WIL2-NS cells, the contracting chromosome loops forming the polar centromere cluster contain the meiotic cohesin REC8 (Figure 3D,E). It appears that this contracting centromere cluster is spatially related to the nucleolar rim and molecularly to the increasing concentration of CYCLIN B1/MOS (Figure 3F,G), as well as with the emerging centrosome astral activity (Figure 3H). These observations on centromere clustering around the nucleolus may be related to the structure described as a ‘karyosphere’ characteristic of oocyte maturation before GV breakdown [70,71], where an extreme microtubule nucleation activity is found essential for the formation of the first meiotic spindle before the start of metaphase I [72]. Finally, figures of very late diakinetic monopolar prophase were observed through α-TUBULIN and DAPI staining with the radial rows of AURKB doublet tandems on the semi-condensed, mostly co-parallel, sets of bivalents preparing for metaphase I (Figure 3I). These oocyte-like cells were documented both in irradiated male and female lymphomas and in DOX-treated breast cancer MDA MB 231 cells (all TP53-mutant). In these models, MOS expression enhanced by DOX was confirmed by qRT-PCR ([31] and not shown). In summary, our observations of 3 TP53-mutant cell lines suggest that, after DNA damage, they undergo a modified polyploid meiotic prophase, including PM and DLM-like setting, with recombination likely occurring between centromere doublets (as synaptonemal complexes and telomere clusters are not observed). The monopolar spindle of DLM figures retains the AURKB attachment and thus centromere pairwise cohesion (where only the poleward partner is attached to the MT as reported [73], thus enabling further reduction division in Met I).

However, observation of the bi-looped monopolar DLM-like figures operated by the proteins of meiotic recombination raises the question of homologue positioning in these polyploid tumour cells.

### 3.3. Parental Group Segregation, Fusion and Homologue Pairing in the PXT Model of PM

**Figure 4:**
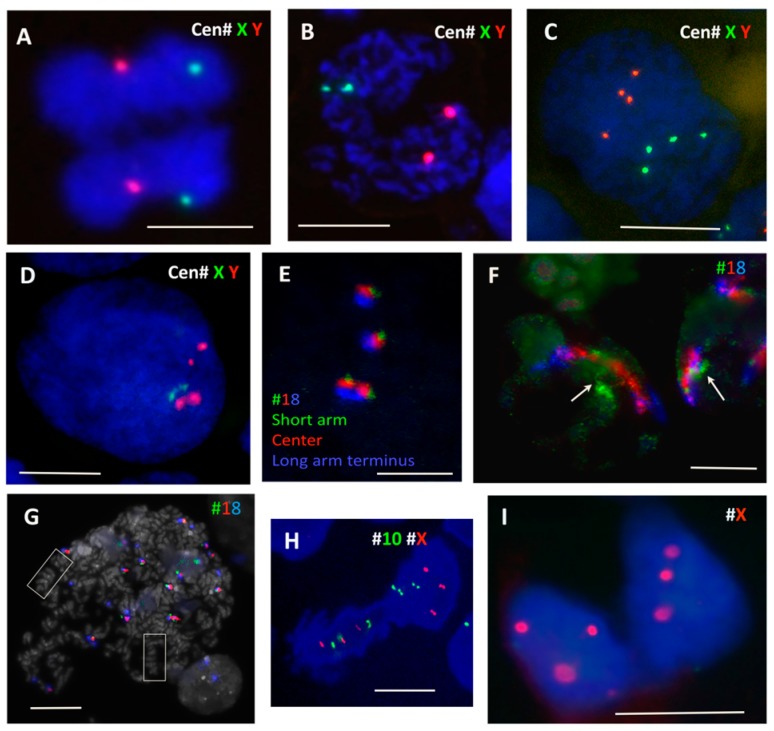
Fluorescence in situ hybridisation (FISH) studies of chromosome positions during polyploidisation and de-polyploidisation in genotoxically treated tumour cells. (**A**–**D**) PXT treatment of male diploid WIL2NS cells reveal the following: (**A**) Separation of both #X (green) and #Y (red) specific centromere labels in anaphase. (**B**) Two X, Y centromere signals each in two connected chromosome groups 19 h after inactivation of the spindle with PXT. (**C**) Four centromeric signals of each sex chromosome opposing each other in an octoploid cell, 44 h after PXT treatment. (**D**) Replicated #X and #Y centromere signals cohesed as dyads, characteristic for 2 days after PXT treatment. (**E**–**G**) Position of homologues and multiple #18 labels revealed by a three-colour (p: green; centre: red; terminus q: blue) painting probe in 10 Gy-treated Namalwa lymphoma cells. (**E**) Control metaphase chromosome 18 after triple labelling. (**F**) Extended territories of #18, touching at green p-ends (arrows) in polygenomic nuclei on 6 days post 10 Gy. (**G**) Large endomitotic polyploid metaphase cell displaying co-parallel orientation of condensed chromosomes (boxed) with 16 territories of condensed chromosome 18 (7 days post-IR). (**H**,**I**) Bipolar division of polyploidised (normally para-triploid) HeLa cells showing on (**H**) the meiosis I-like reduction segregation of three pairs of binemic chromatids for #10 (green) and #X (red). (**I**) Segregation of uni-nemic chromatids as indicated by centromeric signals for #X in HeLa cells (10 Gy, Day 2). Bars equal 10 µM.

To verify the possibility of segregation of parental sets of dyads during PM, two-colour centromeric FISH for #X and #Y centromeres was performed in the diploid male WIL2-NS PTX model. While in the untreated control sample the majority of mitoses showed chromatid separation at anaphase (Figure 4A), at 17–19 h post PXT treatment there was a clear tendency in the PM for each FISH signal doublet (indicating replicated chromatids) to be separated into each chromosome group in about 50% of cells (Figure 4B). As such, this bi-nemic parental segregation was apparently non-random for the sex chromosomes, but at least in the WIL2-NS PXT model it seemed either unstable or, more likely, that a subsequent event had already started in a proportion of the cells. Indeed, after the next round of DNA replication (sampled after 44–48 h of PXT treatment) some 8C DNA interphase nuclei displayed four labels for each sex chromosome, often polarly arranged in an antiparallel fashion (Figure 4C). However, in most re-replicated cells, the centromeric FISH already showed an opposite tendency, with a juxtaposition or even colocalisation of the #X and #Y labels and their centromerically cohesed dyads (Figure 4D). Enumeration revealed the juxta-position of sex chromosomes both in 14–18% of the control and PXT-treated cells after 17–19 h. This fraction nearly doubled (32%) after 48 h post PXT treatment. At this latter time point, there was also a release of univalent sex chromosomes in small nuclear buds indicative of instability and ongoing micronucleation (not shown).

Thus, in the PXT model of PM, the pseudo-mitotic cells very likely spatially separate the replicated parental genomes (or parts thereof) in the first G2-phase post treatment and keep this re-arrangement in the ensuing PM without a mitotically functional spindle. This retains both chromosome groups in the same cell that subsequently becomes tetraploid (graphically represented in Figure 1, right panel, A). After the next replication round, a fusion of parental genomes seems to occur. Thus, the separation of parental genomes followed by their fusion with the signs of homologue pairing should be identified as autokaryogamy, the earliest step in sexual evolution as classified by Raikov [74].

### 3.4. End-To-End Joined Inverted Homologues

To test for homologue positioning, we performed a three-colour FISH painting of chromosome 18 territory labelling with chromosome 18 labelled green, red, and blue to allow for orientation studies. This analysis performed on Namalwa diploid lymphoma cells sampled on untreated control (Figure 4E) and on 6 and 7 days post 10 Gy IR revealed end-to-end or head-to-head linking of extended homologue pairs (Figure 4F), which correspond to the bi-looped endo-prophase undergoing meiotic-type recombination (Figure 3A,B). The head-to-head linked orientation of homologue pairs is compatible with zigzag and co-parallel tandem chromosome orientation, which was observed in some endomitotic cells with condensed chromosomes (Figure 3I and Figure 4G) and is graphically presented in Figure 1, right panel, B.

### 3.5. Linear Bipolar Reduction Divisions Segregate Bichromatids and Then Chromatids

Reduction divisions starting with diplochromosomes (not shown) and metaphase I-like segregation of REC8-cohesed centromeric dyads in a bipolar division presented here on HeLa cells (Figure 4H) has also been observed in IR-treated tetraploid lymphoma cells [38] and by other researchers [75,76,77]. The next division, omitting the S-phase (as earlier described [38]) separating chromatids in an amphitelic manner (like typical mitosis), brings cells to para-diploidy or para-triploidy as in the case presented in Figure 4I, on HeLa (HeLa is normally para-triploid). Such a scenario (graphically presented in Figure 1, right panel, C) may follow co-parallel orientation of sister chromatids and homologues in endo-metaphase if the DNA DSB-induced chiasmata between sisters become released. Usually such cells do not polyploidise over 8C.

### 3.6. Radial Reduction Divisions Can Segregate Whole Genomes

**Figure 5:**
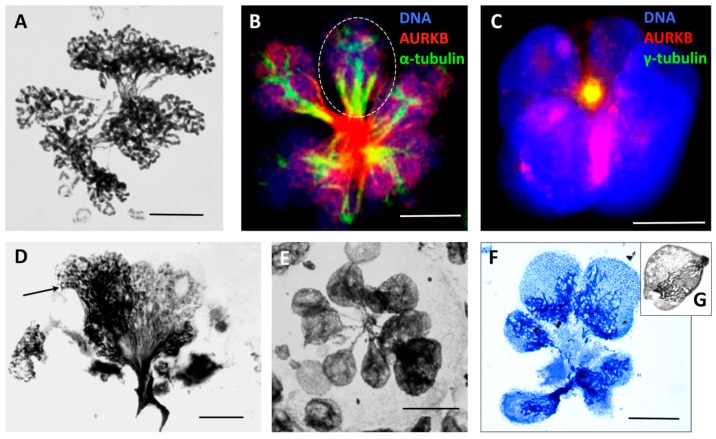
Multipolar metaphase-anaphase cells undergoing radial de-polyploidisation and pedogamic fusions of karyotomy products: (**A**) Multipolar mitosis figure displaying unequal partitioning of bridged sub-nuclei with connected chromosome ends separating circular chromosomes in a HeLa cell (10 Gy, Day 3). (**B**) Multipolar ana-telophase stained for AURKB (red) and a-tubulin (green). The fusion of the division mid-bodies tragged towards one centre and, in addition, the pedogamic fusion of the karyotomy products are seen as three (encircled) or one of the two spindle ‘twigs’ segregating with them, in WIL2-NS (PXT, Day 5). (**C**) A restituted radial telophase converging mid-bodies to the composed centrosome, stained for γ-tubulin and AURKB (colocalised as a yellow spot) in Namalwa cells (6 days post 10 Gy). (**D**) Low resolution EM of a giant cell that has failed radial de-polyploidisation, leading to a bouquet appearance of spindle trapped genomes partly forming sub-nuclei (on the right) and remaining looped (arrowed, on the left) (Ramos lymphoma cell, 14 days post 10 Gy) (reproduced with permission from [24]). (**E**) Multilobed telophase cell after multipolar radial reductional segregation (HeLa cells 5 days post 10 Gy) (reproduced with permission from [78]). (**F**) Radial cytotomy with unequal segregation of the genomic material in cellularised sub-cells. HeLa cells 10 Gy, Day 5 (reproduced with permission from [78]). (**G**) Secondary sub-cell with the meiosis-like ‘bouquet’ arrangement of chromosomes. HeLa cell, 15 days post 10 Gy. (**E**–**G**) Toluidine blue staining. Bars equal 10 µM.

De-polyploidisation leading to the separation of whole genomes was frequently observed in heavily radio-chemotherapy-treated lymphoma, HeLa, and MDA MB 231 cells from the end of the 1st week and onwards, up to 3 weeks [24,55,78,79], with the multiple genomes likely unable to linearise and separate chromatids (Figure 5A). Such multi-genomic polyploid cells (those few that survive the 1st apoptotic crisis) undergo de-polyploidisation by segregating not the chromosomes but the whole genomes, through an incomplete multipolar mitosis, which is sometimes referred to as endomitosis. Indeed, several semi-spindles and often a few bipolar or multipolar spindles simultaneously appear, bridging the products of these untidy karyotomies, which is followed by radial cytotomy as previously described [24,78,80] and graphically presented in Figure 1, right panel, D. The mechanics likely include pulling together the mid-bodies of individual bipolar spindles between the bridged products of karyotomy (unable to undergo abscission) to the centrally localised centrosome (Figure 5B,C). The products of neighbour karyotomies can pedogamically fuse before cytotomy after PXT treatment (Figure 5B, encircled) but also after IR (MS in preparation). From the side, this polyploid endo-telophase may look like a bouquet (Figure 5D), where its origin from the poorly processed bi-looped endo-prophase can be seen (arrow). The multi-genomic endo-telophase is again endowed by a terminal ‘DNA button’ of the converged chromosome bridges (Figure 5E). This button is then dynamically removed by an autophagic vacuole releasing free sub-nuclei (Figure 5F) (detailed in [79,81]). This radial genome segregation often also includes the sequestration and extrusion of separate degraded genomes [24,79]. The process is completed by cellularisation of the subnuclei sequestering an individual cytoplasm with organised cytoskeleton and releasing sub-cells (Figure 5F) [18,24,79]. The resulting daughter cells will either be clonogenic (and often immediately start mitotic divisions) or die, but may attempt another round of meio-mitosis. Moreover, we occasionally found a very high frequency of meiosis-like bouquets in the secondary small cells liberated from polyploid HeLa cells after IR (Figure 5G), while repeated peaks of REC8 transcription (Days 5, 10, and 25) were registered [32] in this model. Alternatively, a blastula-like body is formed from this multinuclear cell, with upregulated nuclear OCT4A and NANOG in the radially arranged sub-nuclei testifying to their embryonal nature [79,81], and this process is favoured by the serum-poor spherogenic medium [82].

## 4. Discussion

In the last decade, cancer genome sequencing has brought us the crushing realisation that cancer is much more complex than previously thought and has highlighted the limitations of the somatic mutation theory of cancer [83,84]. In addition, massive chromothripsis DNA repair cascades associated with polyploidy [7,10] have been revealed in cancer progression adding more intricacy to the cancer aneuploidy paradox.

Experiments detailing the escape of cancer cells from genotoxic challenge indicate two opposite but complementary strategies. Firstly, a very small proportion of cancer cells (<0.1%) still can escape death from seemingly lethal dosage of genotoxic therapy [33,55,60,61,85,86]. They succeed by undergoing reprogramming [20,86] and complex multi-step, reproductive ploidy cycles, which are experimentally observed over a period of weeks. Although nearly all of the cells initially undergo a degree of polyploidisation, very few of them finally survive. They represent rare single cells undergoing reprogramming and complex multi-step, reversible ploidy cycles, which are experimentally observed over a period of weeks. Although these rare ‘statistical outliers’ originating from polyploid cells present serious methodical difficulties for following and fate-mapping, making studies of their nature and genesis complex (particularly as later time-points), they are apparently the major mediators of resistance to many conventional therapies and responsible for relapse [33,55,57,58,59,60,61,62,63,64,65,66,87]. The accompanying reversal of senescence may also be important in this process [58,60,88,89] and even apoptosis reverse may be supportive [90,91].

But the question remains as to how the reproductive minority achieves this improbable escape from genotoxic damage and how this is related to aneuploidy? This process can likely only be explained through the non-linear thermodynamics of unstable systems [92], in which adaptation through chaotic regulations of multi-dimensional transcriptional attractors can occur, exhibited as epigenetic heterogeneity, to adapt such improbable events. The predetermined chaos is a key property of stem cells and cancer cells for cell fate change [93,94,95,96], which is implemented through the clonal and non-clonal [97] selection of survivors. Thus, reprogramming is a key biological instrument of chaotic regulations for the achievement of rare and improbable events and this reprogramming as found [20,59] is coupled to the illicit transition to tetraploidy that occurs after DNA damage in many cancer cells. Therefore, the accompanying genome instability, general mutability, and heterogeneity seems a ‘condition sine qua non’ for the treatment escape of heavily damaged cancer stem cells. Subsequently, quite expectedly, the phenotypic variability of individual cells has been revealed in aneuploidy populations [98].

This same instrument, whole genome duplications with the reprogramming, is evident during the prolonged evolution of prokaryotes and unicellular eukaryotes, providing them with the capacity to adapt and survive seemingly catastrophic events [22,99,100]. The accompanying inevitable cost frequently observed as aneuploidy and cancer is a tribute to adaptive evolution.

However, the same polyploidy-associated reprogramming is also executing an opposing strategy to counter-balance this genomic chaos. Here, the reproductive fidelity of tumour cells is restored through a meiotic-like process, which reduces aneuploidy and genome aberrations. Meiosis in principle needs polyploidy, while somatic meiosis occurs in ploidy cycles [101,102]. In this paper, we focus only on this aspect of reproductive survival of cancer cells.

In this study, we assessed how numerous untreated and DNA-damaged tumour cells polyploidise and segregate their genomes, and we looked for common themes associated with meiosis. We saw that PM commonly occurs with a mitotically disabled spindle and closed telomeres, thereby evading practically all of the checkpoints of the mitotic cell cycle. Parental genome segregations with spindle un-coupling has been suggested by Walen in normal human senescing fibroblast cultures and in those treated with spindle poisons, and extrapolated to stem cell biology, highlighting haploidy as an operating unit of genome segregations [103,104,105]. Such metaphases ‘exploded’ by nocodazole treatment or spontaneously by stress [106] and segregation of haploid genomes [107] have also been reported. PM paves the way for tumour cells to tolerate otherwise fatal amounts of DNA damage, thereby escaping immediate cell death. At the same time, end-to-end linkage of replicated chromosomes segregated as two parental genomes leads to the establishment of tetraploidy (very likely after mutagenic translesional DNA synthesis, allowing them to tolerate the DNA damage [108]). Nevertheless, the resulting genome instability, aneuploidy, and mutagenesis ultimately require compensation for the sake of reproductive fidelity. Segregation by PM of parental genome sets, by-passing the spindle checkpoint, is the first step towards this objective: its anti-parallel orientation likely pre-determines the conjugation of homologues that follows. The process of the primitive life-cycle in one cell is thus initiated, perhaps shedding light on the evolutionary origin of sex, which doubles the amount of DNA, and meiosis which halves it. Somatic tumours are naturally expected to undergo asexual ploidy cycles. The mathematical studies of Kondrashov showed that asexual ploidy cycles and recombination between sisters decreases the mutational load of aneuploidy, and these cycles should have preceded and ‘facilitated the origin of sex, by providing a means of orderly genetic reduction, available immediately after the origin of syngamy’ [101]. We have observed these processes in various human cancer cell models (lymphomas, ovarian germline cancer, breast cancer, and cervical cancer), particularly provoked by genotoxic stress (ionising irradiation, etoposide, and doxorubicin) and mimicked by PXT through spindle inactivation, where segregation of parental genomes as chromosome dyads within the same cell results in tetraploidy. However, this tetraploidy is not a simple endoreduplication, it is unusual and the way it is achieved in cancer stem cells (by separation followed by fusion and conjugation of parental genomes) heralds the initiation of a life-cycle. Attention should be paid to the telomere associations that accompany the PM. On one side, telomere bridges can be due to telomere erosion and favour their deletion [109]. However, in a reproductive context where the telomere label is preserved [28], it is more likely that the formation of a ring-shaped chromosome facilitates end-replication and prevents telomere erosion.

Subsequently, persisting DNA DSB facilitate extended (bi-looped) endo-replicated chromosomes in a peculiar endo-prophase arrangement with clear meiotic features. The peculiarity of this meiotic endo-prophase is the absence of the telomere cluster and synaptonemal complexes (SCs), the hallmarks of conventional meiosis. To our knowledge, despite the abundance of SCP3 in the cytoplasm, SCP3-positive SCs have not been found in tumour cells [39,110]. Nevertheless, the features testifying to the meiosis-like recombination events are evident. It seems that, while in the G2, preceding PM, recombination is occurring at the telomere ends and between sister and nonsister arms, at the next stage of the looped endo-prophase, it may be preferentially occurring on kinetochore proteins between centromere doublets. This can currently only be deduced from our observations of rare cells where centromere/kinetochore tandems are found colocalised and intermittent with DMC1 and RAD51/γH2AX, as reported previously [38]. In turn, centromere doublets are colocalised with REC8, and in turn the foci of DMC1 colocalised to DNA DSBs marked by γH2AX are tandemly located along the looped mono-polar REC8/MOS organised endo-prophase. It can be suggested that MOS, which is capable of phosphorylating the microtubules of spindles [111] is participating in the regulation of this process. Moreover, in polyploid tumour cells, we found structures resembling karyospheres (karyosomes) of maturing oocytes acting in cooperation with REC8 and MOS in the formation of the centromere cluster around and then beside the nucleolus. Interestingly, this structure nucleates (through MOS) a monopolar aster, described as an initial step in setting the first meiotic spindle [72]. It can be also logically hypothesised that the DNA ‘button’ between segregated paternal sets in mitotically disabled PM (depicted on Figure 1, right panel, A) is composed of the nucleolar-organising acentric chromosomes and represents a nucleating centre of the future monopolar spindle and karyosphere of late meiotic oogenic prophase. Following recombination DNA repair and reduction, the meiosis-like process can return cells to the mitotic cycle after a formerly fatal DNA damage insult, thus locking together the two reciprocal parts of the cancer cell life cycle, proliferative and reproductive (Figure 1, left panel). As such, p53 as the guardian of the genome has the role of preventing this error-prone process (although germline cancer cells such as PA1, reflective of cancer stem cells, may be the exception).

It is no surprise that our studies of the ploidy cycles of genotoxically stressed somatic tumours have drawn upon the early evolutionary forms of meiosis and apomixis [45,46,48,49]. In particular, in the absence of SCs, DNA recombination repair can start between the satellite DNA of the two cohesed centromeres. This kind of meiotic recombination is thought to have evolved earlier than that mediated through SCs. What is actually synapsed and recombined, sisters and likely also homologues remains to be clarified. SCs evolved as part of conventional meiosis in early eukaryotes much later than the mechanism of recombination DNA repair in bacteria. It is possible that tumour cells, whose gene expression profile reverts towards the earlier eukaryotic phylogenetic strata [15,16,22], use the recombination variant of synapsis between homologs on very short SCs between kinetochores and recombines on satellites of sister chromatids. In species lacking SCs, the cohesin complexes themselves were suggested to assemble the recombination machinery. In these cases, Maguire suggested the presence of rudimentary small SCs located between cohesed centromeres and pointed out that REC8 conserved through evolution since the proto-eukaryotes [45,112] may be involved, which we consistently found in tumours at these same sites. In particular, it is possible because the mitotic G2 DNA damage checkpoint and recombination checkpoint of meiotic prophase have similar molecular regulators [113], allowing DNA damaged tumour cells to glide from mitotic G2 arrest into meiotic prophase [60]. In turn, the observed monopolar spindle of DLM-like figures, preserving the attachment of kinetochores to centromere doublets in this endo-prophase, apparently takes care both for recombination and reduction division, the main purpose of meiosis. Some authors [114,115] using SC-null mice revealed that the meiotic chromosomal core consisting of the cohesin complex recruits DNA recombination proteins and promotes synapsis in the absence of axial elements in mammalian meiotic cells. The end-to-end pairing of homologues known in ‘reverted meiosis’ is observed in holocentric species [116], while end-to end joining of dyads is seen during meiosis of autopolyploids [117]. Our data are premature for further discussion of these specific issues. Irrespective of how recombination occurs, the DNA damage can be repaired by homologous recombination through polyploidy [118], and some gene conversion (reversing mutation) is thus possible.

From a mechanistic point of view, the observed genome segregation through end-to-end linked dyads (first described by Grell [119] in *Aulacantha scolimantha*) seems the only plausible means of genome preservation (by-passing the spindle checkpoint), while the end-to-end connection of dyads by inverted homologs allowing for flexible co-parallel orientation provides the most convenient way for pairing, recombination, and then reduction division, keeping sister centromeres throughout together (see Figure 1A–C, right).

The other tactic employed by damaged tumour cells is the cellularisation and segregation of whole genomes/sub-cells by radial cytotomy. This process recorded by video-cinematography was termed ‘neosis’ [57] and is compared to sporogenesis [61]. This mechanism of radial de-polyploidisation is agnostic towards aneuploidy and can partition different numbers of genomic complements into cellularised sub-nuclei. As such, the radial de-polyploidisation performed through chromosome bridges also neglects the spindle checkpoint and the issues associated with aneuploidy. Together with the accompanied pedogamic genome re-assortment, this kind of de-polyploidisation may be one of the main causes of chromothripsis in human cancer cells.

However, the stage depicted in Figure 1D (right) may have in fact another biological ‘purpose’. Radial cytotomy perpendicular to karyotomic axes, leaving cytoplasmic bridges between sub-cells is a characteristic feature of the early embryo cleavage stages. Creating an ordered architecture of a blastula for presumptive diversification of ecto- and endoderm has been well described in Volvox [120]. Interestingly, polyploid melanoma cells capable of initiating tumour growth arrange their sub-nuclei in a similar, strictly radial manner [121]. The literature data indicate that a mature multinuclear tumour cell (Day 6–7 post-damage) can convert into a blastula-like embryoid (particularly if it is grown in a serum-free spherogenic medium) and that even a single multi-nuclear tumour cell can propagate a tumour in vivo [82,85]. These data are fully in accord with the more than a century-old embryological theory of cancer [81,82,110]. It seems that different stages of tumour growth and response to therapy not only involve reciprocal parts of a cancer life-cycle (joined by asymmetric division (Figure 1A,C, left panel) but also cycle on their own, with a different speed according to environmental conditions and the amount of genotoxic stress.

## 5. Conclusions

We propose the presented mechanisms, albeit incomplete, as an evolutionary primitive (a)-sexual process adapting to environmental challenges, which can explain key observations underlying the therapeutic resistance of cancer cells and the aneuploidy paradox.

## Figures and Tables

**Table 1:** Primary antibodies and their source.

	Description	Specificity/Immunogen	Used Concentration	Product No. and Manufacturer
AURORA kinase B	Rabbit polyclonal	Peptide derived from within residues 1–100 of Human Aurora B	1:300	ab2254, Abcam, Cambridge, UK
Bromo-deoxy-uridine BrdU	Mouse monoclonal	Reacts with 5-bromo-2-deoxyuridine	1:200	A21300, Invitrogen, Carlsbad, CA, USA
Centromere Protein	Human	Derived from human CREST patient serum	1:50	15-234, Antibodies Inc, Davis, CA, USA
Serine/thre onine protein kinase CHK2 (phospho T68)	Rabbit polyclonal	Epitope around the phosphorylation site of Threonine 68 (VSTpQE) of human Chk2	1:100	ab38461, Abcam, Cambridge, UK
CYCLIN B1	Mouse monoclonal	Raised against a recombinant protein corresponding to human cyclin B1	1:100	sc-245, Santa Cruz, Dallas, TX, USA
DMC1	Mouse monoclonal	Specific for DMC1—does not cross-react with the related protein Rad51	1:100	ab11054, Abcam Cambridge, UK
LAMIN B1	Goat polyclonal	Peptide mapping at the C-terminus of Lamin B1 of human origin	1:200	sc-6216, Santa Cruz, Dallas, TX, USA
MOS (C237)	Rabbit polyclonal	Epitope mapping at the C-terminus	1:50	sc-86, Santa Cruz, Dallas, TX, USA
NUMA	Mouse monoclonal	N-terminus region of human NuMA	1:50	107-7, Calbiochem, Merck, Burlington, MA, USA
γ-H2AX	Rabbit polyclonal	Recognises mammalian, yeast, *D. melanogaster*, and *X. laevis* γ-H2AX	1:200	4411-PC-020, Trevigen, Gaithersburg, MD, USA
REC8 (E-18)	Polyclonal goat	Peptide mapping near the N-terminus of Rec8 of human origin.	1:50	sc-15152, Santa Cruz, Dallas, TX, USA
α-Tubulin	Mouse monoclonal	Epitope at the C-terminal end of the α-tubulin isoform in a variety of organisms	1:1000	T5168, Sigma-Aldrich, St. Louis, MO, USA

**Table 2:** The enumerations of CM and PM mitoses in ETO-treated PA1 cells over 19 days.

PA1	Mitotic Cells Counted	Normal Metaphases (CM) %	PM Metaphases (PM) % of CM	Anaphase + Telophase % (CM)	M/A + T (CM)
ctrl	50	82	12	18	5
ETO d7	53	85	11	15	6
ETO d19	55	67	24	33	2
